# Anniversary Discourse before the New York Academy of Medicine

**Published:** 1848-06

**Authors:** 


*Anniversary Discourse before the New York Academy of Medicine. Delivered in the Broadway Tabernacle, November 10th*, 1847. By John W. Francis, M. D.

This is a long discourse for an anniversary speech; it occupies, in fact, an entire pamphlet of one hundred and twelve goodly sized pages. This lengthened space has allowed the distinguished orator to introduce a vast variety of topics, medical, philosophical, biographical, historical, &c., more or less remotely connected with the present and past state of our profession, especially in the city and State of New York. All the great physicians of that commonwealth, from "Johannes Megapolensis, and his son Samuel," of the ancient "Dutch doctors," down to "Manly, Roberts, Gardner, Wood, and others," of the present day, are duly praised. We have seldom seen a production of the kind which displayed more goodness of heart and irrepressible vivacity. The amiable author seems to have been intent on praising everybody and almost everything, and few who read his sprightly pages will suspect that so much warmth of feeling comes from one whose head has been frosted by so many winters. While thus expressing our admiration of the generous enthusiasm and excellent temper of our author, we should be glad to be able to speak as approvingly of some of his sentiments.

"An Academy of Medicine in this city," (New York,) the author remarks, "was a moral necessity; it was demanded by the daily increasing perversion of a noble science, by the sullied dignity of an honorable vocation, by the predominance of evils of saddest issue, and by the long-neglected claims of injured humanity." We cannot help feeling regret whenever we hear our profession spoken of in such terms, and especially by those who are distinguished in its ranks. It is indeed quite too common in our own household, to speak of it as degraded, corrupt, vulgar, &c. This is all wrong,—unjust, and impolitic.

That a great majority of the practitioners of the United States, are better instructed and much more enlightened and safe practitioners than the physicians of thirty or forty years ago, when the profession was not so decried, we have no doubt; neither have we any doubt that the continued declamations of the less successful, and therefore dissatisfied members, against its honour and dignity, is a principal cause of the use of such language by the laity. If the people prefer quacks to regular practitioners, we are no otherwise accountable than as we encourage it by defaming our own brotherhood, or by individual ignorance and neglect bring our art into disrepute. It is not to be expected, however, under any circumstances, that the mass of the people can become sufficiently acquainted with the principles of our science to rightly appreciate its excellence, or sufficiently intelligent to preserve them from the machinations of unprincipled knaves. The weakness and credulity on which empirics thrive belong to the human mind, and can never be erased by any teaching, not even of experience, and far less by any course of ours. The "doubters and disputers of our progress," to use the author's own language, have not "compared" our science of the present day with its condition thirty years ago, or they would exult in its advancement, rather than whine over the supposed decadence of the profession. In our own country, we need only point to the array of authors and their achievements, supplied by Dr. Francis himself, to be convinced that our progress is onward. By the way, in this enumeration we find some strange collocations. Thus, while Holmes, Goddard, &c., are classed among the *authors* of works, Dunglison, Morton, Meigs, &c., are ranked only with *editors!*

We are in no mood, however, to find fault with our venerable brother for the little inaccuracies of this sort to be observed in his dissertation. The ardent patriotism, the love of our science, the admirable temper, added to the many judicious remarks and interesting reminiscences which it contains, present too many claims to our thanks to allow even of just criticism. We are persuaded that all who have read Dr. Francis's discourse will join us in this acknowledgment of its merits.

